# Inappropriate urine culture testing in nursing home residents: a driver of antibiotic overtreatment and antimicrobial resistance

**DOI:** 10.3389/fmed.2026.1931709

**Published:** 2026-08-17

**Authors:** Bijou Muke Mbaya, Manon Mbangi, Nodia Nzale Kosia, Judith Ahondju Dende, Lionel Hemut Loïc Mevo Sonagnon, Gloire Mazita Bovy-Lumingu, Arriel Makembi Bunkete

**Affiliations:** 1 EHPAD Bellevue, Bourges, France; 2 Department of Nephrology-Dialysis, University Hospital of Guyane, French Guiana, France; 3 EHPAD Anna Quinquaud, Guéret, France

**Keywords:** antibiotic overuse, antimicrobial resistance, asymptomatic bacteriuria, nursing homes, urinary tract infection

## Abstract

**Background:**

Inappropriate urine testing and antibiotic prescribing are common challenges in nursing home residents evaluated for suspected urinary tract infection (UTI). This study evaluated urine testing practices, microbiological findings, and antibiotic prescribing in two French nursing homes.

**Methods:**

We conducted a retrospective observational study of 102 nursing home residents evaluated for suspected UTI between June 2024 and April 2026. Demographic, clinical, microbiological, and therapeutic data were retrospectively collected. Antibiotic prescriptions were assessed according to French Infectious Diseases Society (SPILF) criteria. Descriptive analyses were performed.

**Results:**

The mean age was 83.0 ± 8.3 years, and 71 (69.6%) residents were women. Urine dipstick testing was performed in 95 (93.1%) residents and was positive in 87 (91.6%). Urine cultures were performed in 87 residents, of whom 60 (69.0%) had positive cultures. ESBL-producing Enterobacterales were identified in 31 (51.7%) positive urine cultures. Twenty-seven residents received empirical antibiotics despite a subsequently negative urine culture, and 42.5% of antibiotic prescriptions were classified as inappropriate according to SPILF criteria. Fosfomycin was the most frequently prescribed antibiotic. Non-specific geriatric manifestations were common, highlighting the diagnostic difficulty of distinguishing symptomatic UTI from asymptomatic bacteriuria.

**Conclusion:**

Inappropriate urine testing and antibiotic prescribing were frequent in this nursing home population. Positive urine cultures, including those yielding ESBL-producing organisms, should not be interpreted in isolation as evidence of symptomatic infection or an indication for antibiotic therapy. Strengthening diagnostic stewardship and adherence to clinical criteria may reduce unnecessary urine testing and antimicrobial exposure while promoting appropriate antibiotic use.

## Introduction

Urinary tract infections (UTIs) are among the most common bacterial infections in older adults and represent one of the leading indications for antibiotic prescription in nursing homes (1, 2). As populations age and the number of institutionalized older adults continues to increase worldwide, optimizing the diagnosis and management of suspected UTIs has become a major public health priority (3). In long-term care facilities, UTIs account for a substantial proportion of healthcare-associated infections and contribute considerably to antibiotic consumption, healthcare costs, and hospital transfers (4, 5). Despite their apparent frequency, distinguishing true infection from asymptomatic bacteriuria remains one of the greatest challenges in geriatric medicine (6).

The diagnosis of UTI in older adults is particularly difficult because clinical presentation often differs from that observed in younger patients (7). Classical urinary symptoms—including dysuria, urinary frequency, urgency, suprapubic pain, or flank tenderness—may be absent, attenuated, or difficult to assess in residents with cognitive impairment (8). Conversely, non-specific manifestations such as confusion, functional decline, falls, anorexia, fatigue, or behavioral disturbances are frequently attributed to urinary infection despite their poor diagnostic specificity (9, 10). These atypical presentations often prompt urine testing in the absence of convincing urinary symptoms, thereby increasing the likelihood of detecting asymptomatic bacteriuria rather than clinically significant infection (11).

Asymptomatic bacteriuria is highly prevalent among institutionalized older adults, affecting up to 50% of women and 40% of men living in nursing homes (12). Current international guidelines strongly recommend against screening for or treating asymptomatic bacteriuria in this population, except in a few well-defined clinical situations such as pregnancy or before invasive urological procedures (13–15). Randomized controlled trials have consistently shown that treating asymptomatic bacteriuria fails to improve survival or prevent symptomatic infection while unnecessarily exposing patients to adverse drug reactions, Clostridioides difficile infection, drug interactions, colonization with multidrug-resistant organisms, and increasing antimicrobial resistance (13, 16–18).

The excessive use of urine dipsticks and urine cultures is a major contributor to inappropriate antibiotic prescribing in nursing homes (19). Because bacteriuria and pyuria are extremely common in frail older adults, indiscriminate urine testing inevitably yields positive microbiological results that are frequently misinterpreted as evidence of infection (20). Consequently, the diagnostic process itself may promote antibiotic overtreatment. Several antimicrobial stewardship initiatives therefore recommend restricting urine cultures to residents presenting with compatible urinary symptoms or systemic signs of infection after excluding alternative diagnoses (14, 21, 22). The minimum clinical criteria proposed by Loeb et al. remain one of the most widely adopted decision tools for guiding urine testing and empirical antibiotic initiation in long-term care facilities (21).

Despite these recommendations, inappropriate urine culture prescribing remains common worldwide (19, 23–25). Urine cultures are frequently requested for isolated cloudy or malodorous urine, confusion, falls, or behavioral changes despite the absence of urinary symptoms (24, 25). Such practices contribute to unnecessary antibiotic exposure and promote the emergence and dissemination of multidrug-resistant organisms in nursing homes (18, 26).

In this context, we conducted a retrospective study in two French nursing homes to evaluate current practices for prescribing urine cultures among residents with suspected urinary tract infections. The objectives were to describe the clinical indications leading to urine culture requests, characterize microbiological findings and antibiotic prescriptions, and assess the potential contribution of inappropriate urine culture testing to antibiotic overtreatment and antimicrobial resistance.

## Methods

### Study design and setting

This retrospective observational study was conducted in two French nursing homes (EHPADs) to evaluate practices for prescribing urine cultures and the management of suspected urinary tract infections (UTIs) in older residents. The study covered a 22-month period from June 2024 to April 2026. The participating facilities comprised 190 long-term care beds (120 and 70 residents, respectively) and provided medical and nursing care for highly dependent older adults.

### Participants

**FIGURE 1:**
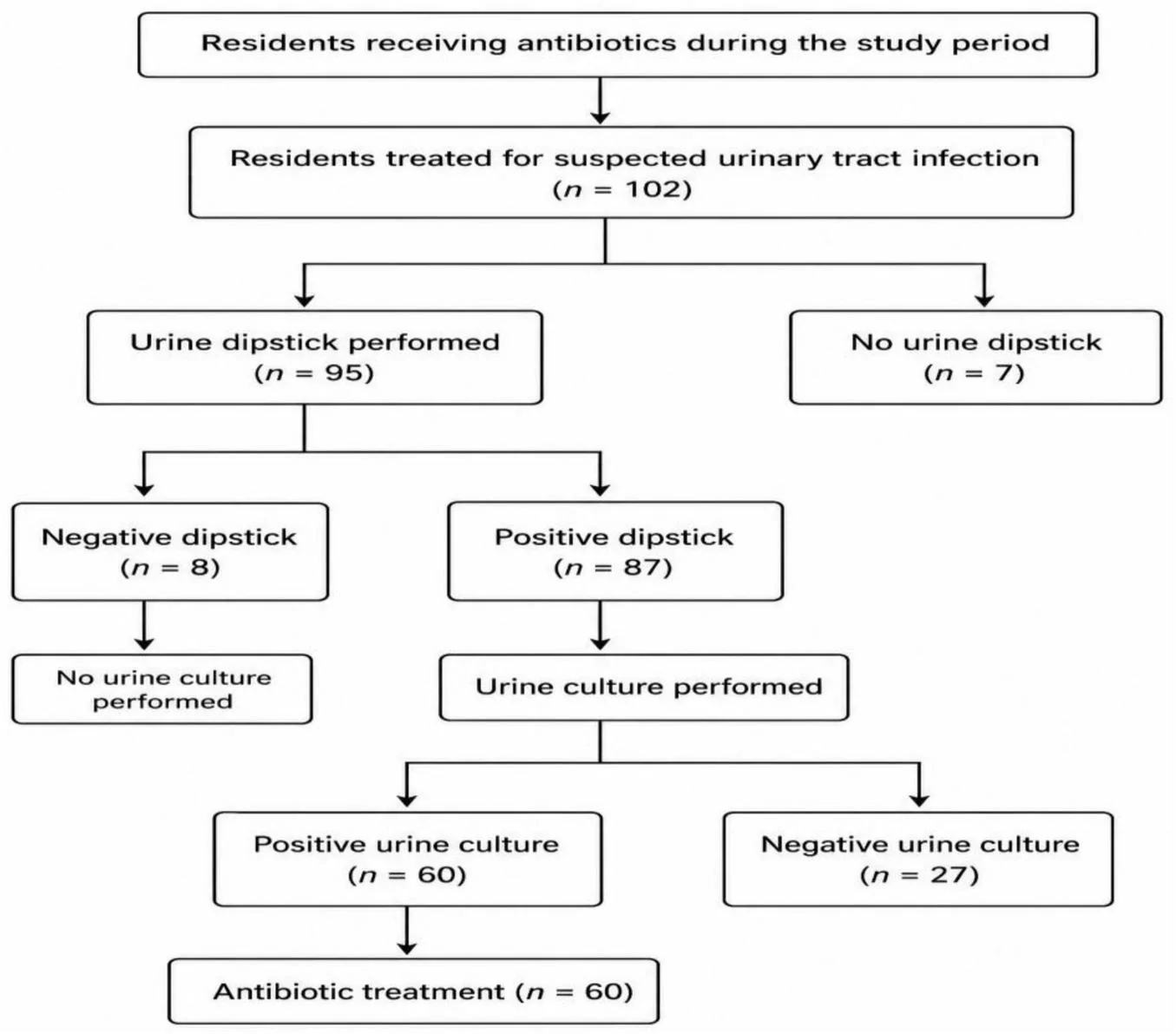
Study flowchart.

All nursing home residents evaluated for suspected UTI during the study period were considered for eligibility. The participant selection process is presented in Figure 1. Cases were identified through pharmacy dispensing records and subsequently confirmed by reviewing the electronic medical records. Residents meeting the predefined eligibility criteria and for whom sufficient clinical, microbiological, and therapeutic information was available were included in the final study population. Residents with incomplete medical records for the variables of interest were excluded. A total of 102 residents met the eligibility criteria and were included in the analysis. For these 102 residents, variables not documented in the medical records were treated as missing and were not imputed; analyses were performed using the available data for each variable.

### Data collection

Data were retrospectively extracted from the electronic medical record system (TITAN^®^) using a standardized data collection form.

The following demographic variables were collected: age, sex, and functional dependency assessed using the Groupe Iso-Ressources (GIR) classification.

Clinical variables included the macroscopic appearance of urine (cloudy, malodorous, or gross hematuria), urinary symptoms (dysuria, urinary frequency, urgency, burning micturition, suprapubic pain, flank pain, acute urinary retention, and recent onset of urinary incontinence), general signs (fever, chills, hypothermia, and deterioration of general condition), and non-specific geriatric manifestations such as acute confusion, falls, somnolence, anorexia, and worsening behavioral disorders.

Additional variables included the presence of an indwelling urinary catheter, urine dipstick results, urine culture findings, isolated microorganisms, antimicrobial susceptibility profiles, the healthcare professional requesting microbiological investigations, the empirical antibiotic prescribed, and any subsequent modification of antimicrobial therapy according to microbiological results.

### Definitions

Definitions of urinary tract infection, asymptomatic bacteriuria, urinary symptoms, complicated infection, and microbiological criteria were based on the recommendations of the French Infectious Diseases Society (SPILF) in force during the study period.

Clinical assessment was based on the presence of documented urinary symptoms and/or compatible systemic signs according to the SPILF recommendations applicable during the study period. Non-specific geriatric manifestations, including acute confusion, falls, somnolence, anorexia, or functional decline, were not considered sufficient, in isolation, to establish a diagnosis of symptomatic UTI. These manifestations were interpreted in the context of the overall clinical presentation and, when documented, alternative potential causes were considered.

A positive urine culture was defined according to the microbiological criteria for significant bacteriuria established by the microbiology laboratory and applicable SPILF recommendations. The presence of bacteriuria and leukocyturia was interpreted in conjunction with the clinical presentation and was not considered, in isolation, sufficient to establish symptomatic UTI, given the high prevalence of asymptomatic bacteriuria and pyuria among nursing home residents. Thus, microbiological findings were not considered an isolated basis for diagnosing symptomatic UTI or initiating antibiotic therapy.

Urine specimens were processed according to routine microbiological procedures. Quantitative urine cultures were performed, and bacterial identification was carried out using matrix-assisted laser desorption/ionization time-of-flight mass spectrometry (MALDI-TOF MS) from isolated bacterial colonies. Antimicrobial susceptibility testing was performed using routine laboratory methods and interpreted according to EUCAST clinical breakpoints applicable during the study period. ESBL-producing Enterobacterales were identified using standard phenotypic screening and confirmation methods according to routine microbiological laboratory procedures.

Antibiotic overtreatment was defined as the initiation of antibiotic therapy in residents who did not meet the SPILF clinical criteria for symptomatic UTI, including situations in which non-specific geriatric manifestations were the only documented clinical findings or in which microbiological findings were interpreted as evidence of infection in the absence of compatible clinical criteria.

### Outcomes

The primary outcome was the description of urine testing and antibiotic prescribing practices among nursing home residents managed for suspected UTIs.

Secondary outcomes included the proportion of positive urine dipsticks and urine cultures, the microbiological distribution of isolated pathogens, the prevalence of multidrug-resistant organisms, antibiotic prescribing patterns, and the proportion of residents receiving antibiotic therapy despite a negative urine culture or in the absence of clinical criteria compatible with symptomatic UTI.

### Bias

To reduce selection bias, all consecutive residents who met the predefined eligibility criteria during the study period were included. Information bias was minimized through systematic review of electronic medical records using predefined variables and standardized data extraction procedures. Owing to the retrospective design, only routinely documented clinical and microbiological data were available for analysis.

### Study size

No formal sample size calculation was performed because the study included all consecutive eligible residents identified during the predefined study period.

### Statistical analysis

This study was designed as a descriptive observational study. Continuous variables are presented as means with standard deviations or medians with interquartile ranges, as appropriate. Categorical variables are expressed as frequencies and percentages. No multivariable analyses were performed to evaluate factors associated with inappropriate antibiotic prescribing or antimicrobial resistance. The study was primarily designed to describe urine testing practices, microbiological findings, and antibiotic prescribing in routine nursing home care. In addition, the relatively small sample size (*n* = 102), the limited number of outcome events, and the lack of systematically collected information on several potentially relevant confounding factors limited the feasibility and interpretability of multivariable modeling. The analysis was therefore intentionally descriptive, and the observed findings were not interpreted as causal associations.

### Ethical considerations

The study was conducted in accordance with the ethical principles of the Declaration of Helsinki and French regulations governing retrospective observational studies. The study complied with institutional and national regulations. Data were anonymized before analysis to ensure participant confidentiality. This retrospective study used routinely collected healthcare data and involved no modification of patient management; therefore, no intervention was performed for the purposes of the study.

## Results

### Study population

During the 22-month study period, 102 nursing home residents were identified as eligible according to the predefined selection process and received antibiotic therapy for suspected urinary tract infection. The mean age was 83.0 ± 8.3 years (median, 85.0 years; IQR, 79.0–89.8). The cohort included 71 women and 31 men, corresponding to a female-to-male ratio of 2.3. Most residents were highly dependent, with 32 (31.4%) classified as GIR 1 and 54 (52.9%) as GIR 2, indicating that 84.3% of the study population had severe functional dependency (Table 1).

**TABLE 1:** General characteristics of the study population.

Variable	Mean (SD)	Median (IQR)
Age, years	83.0 (8.26)	85.0 (79.0–89.8)
	**N**	**%**
Sex		
Female	71	69.6
Male	31	30.4
GIR classification		
GIR 1	32	31.4
GIR 2	54	52.9
GIR 3	3	2.9
GIR 4	13	12.7
GIR 1–2	86	84.3
Total population	102	100

GIR (Groupes Iso-Ressources) is a functional dependency scale used to assess autonomy, ranging from GIR 1 (complete dependence requiring continuous assistance) to GIR 6 (full independence). Lower scores indicate higher dependency.

### Urine dipstick and urine culture testing

Among the 102 nursing home residents included in the study, urine dipstick testing was performed in 95 (93.1%). Eight residents (8.4%) had a negative urine dipstick result, and no urine culture was subsequently requested, consistent with the high negative predictive value of urine dipstick testing for ruling out urinary tract infection in this setting. Seven residents (6.9%) did not undergo urine dipstick testing. Among the 95 residents who underwent dipstick testing, 87 (91.6%) had a positive result and subsequently underwent urine culture according to routine clinical practice (Figure 1).

### Urine culture results

**FIGURE 2:**
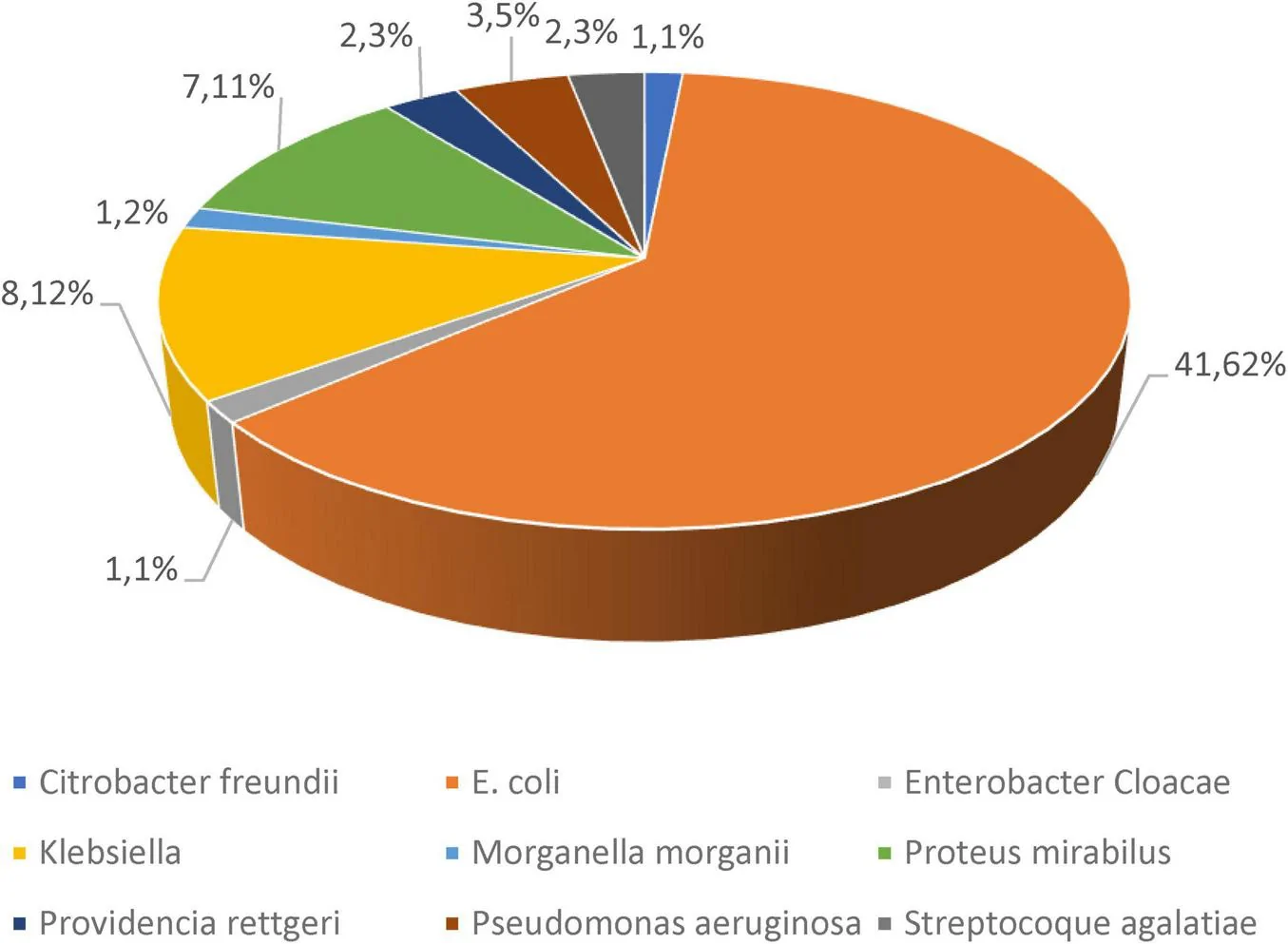
Distribution of bacterial species isolated from positive urine cultures.

**FIGURE 3:**
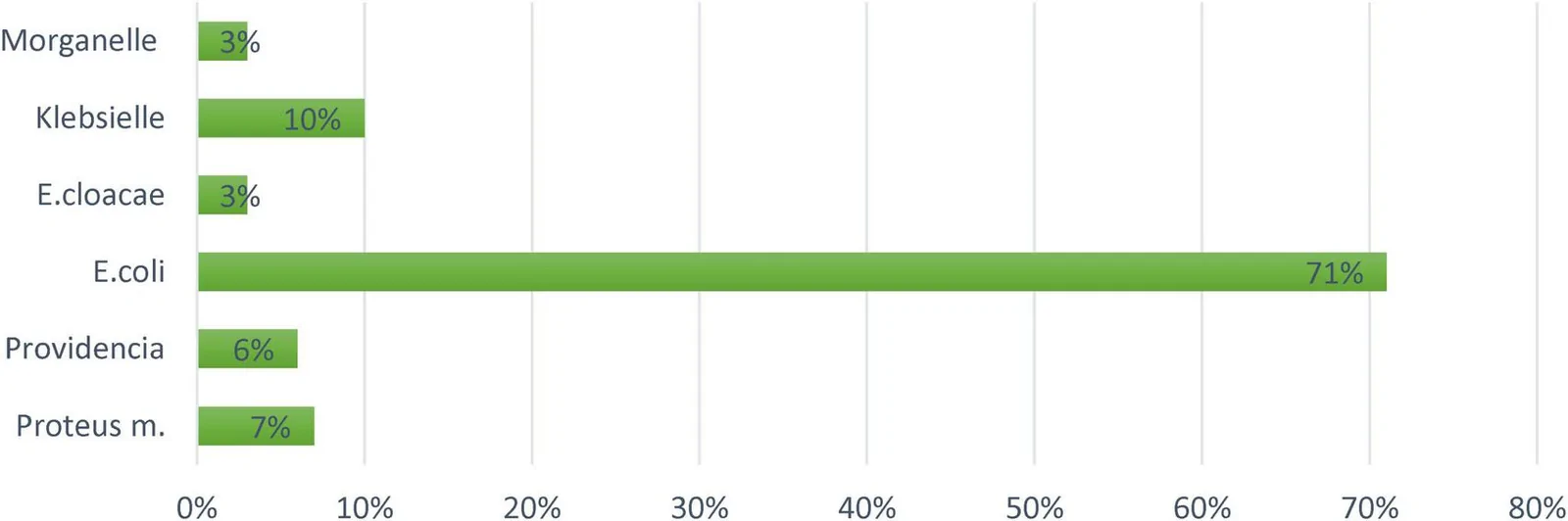
ESBL-producing Enterobacterales among positive urine cultures.

Sixty urine cultures were positive, all showing significant bacteriuria (≥10^5^ CFU/mL), with associated significant leukocyturia (≥10^4^ leukocytes/mL). Among the 87 urine cultures performed, 60 (69.0%) were positive and 27 (31.0%) were negative (Table 2). Enterobacterales accounted for the majority of isolates, with *Escherichia coli* representing more than one-third of all identified microorganisms. Other isolated pathogens included *Klebsiella* spp., *Enterobacter cloacae*, *Proteus mirabilis*, *Morganella morganii*, *Providencia rettgeri*, *Citrobacter freundii*, *Pseudomonas aeruginosa*, and *Streptococcus agalactiae* (Figure 2). Among residents with positive urine cultures, 31 (51.7%) harbored extended-spectrum β-lactamase-producing Enterobacterales (ESBL-E), highlighting the substantial burden of multidrug-resistant organisms in this nursing home population (Figure 3).

**TABLE 2:** Urine testing and microbiological findings.

Variable	*N*	%
Urine dipstick performed	95	93.1
Positive urine dipstick	87	91.6
Negative urine dipstick	8	8.4
No urine dipstick	7	6.9
Urine culture performed	87	85.3
Positive urine culture	60	69.0
Negative urine culture	27	31.0
ESBL-producing isolates	31	51.7

### Clinical presentation

Among the 60 residents with positive urine cultures, 50 (83.3%) presented with at least one urinary symptom associated with general and non-specific manifestations. In contrast, 10 residents (16.7%) presented exclusively with non-specific geriatric manifestations without documented urinary symptoms.

### Antibiotic prescriptions

**FIGURE 4:**
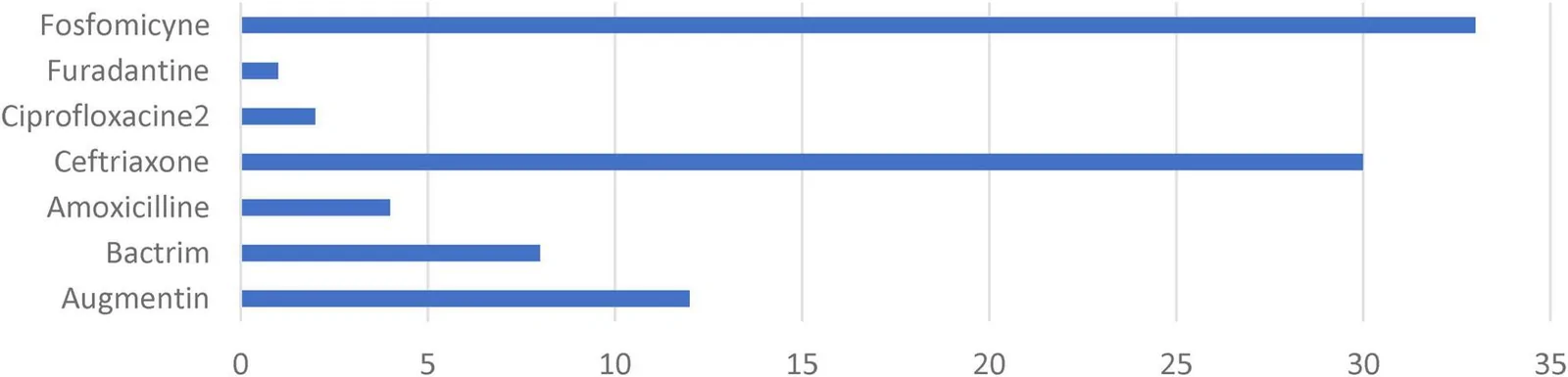
Antibiotic prescription.

All residents with positive urine cultures received antibiotic therapy. Fosfomycin was the most frequently prescribed antimicrobial agent, followed by nitrofurantoin, ciprofloxacin, ceftriaxone, amoxicillin, trimethoprim-sulfamethoxazole, and amoxicillin-clavulanate. Antibiotic selection was guided by the narrowest effective antimicrobial spectrum and adapted whenever possible according to antimicrobial susceptibility testing (Figure 4). Among women with a positive urine dipstick, 27 received empirical antibiotic therapy for suspected cystitis before urine culture results became available. All corresponding urine cultures were subsequently negative, and alternative diagnoses, including dehydration or other infectious conditions, were ultimately identified. Overall, 42.5% of antibiotic prescriptions were considered inappropriate because treatment was initiated despite the absence of clinical criteria consistent with symptomatic urinary tract infection, including residents with atypical manifestations alone or negative urine cultures. In addition, seven residents with severe neurocognitive disorders received empirical antibiotic therapy because urine sampling could not be performed due to refusal or inability to obtain an appropriate specimen.

## Discussion

This study highlights a high rate of urine culture testing and frequent discordance between microbiological results, clinical presentation, and antibiotic prescribing in nursing home residents with suspected urinary tract infection. Overall, our findings demonstrate that urine testing practices are strongly associated with antibiotic overuse and may contribute to the selection of multidrug-resistant organisms in this vulnerable population. However, given the retrospective observational design of the study, these findings should be interpreted as associations and should not be considered evidence of a direct causal relationship between urine testing practices and antimicrobial resistance.

In our cohort, urine cultures were performed in 87 of 95 residents with a positive dipstick result, and 60 (69.0%) were positive (Table 2). However, all positive cultures were associated with bacteriuria and leukocyturia, findings that are common in elderly institutionalized patients and do not necessarily indicate infection (6, 11, 20). Asymptomatic bacteriuria remains highly prevalent in nursing home residents and should not be treated in the absence of compatible clinical criteria (12–15). The high rate of urine culture positivity observed in this study therefore likely reflects colonization rather than true infection in a substantial proportion of cases. Importantly, a positive urine culture, including one yielding an ESBL-producing organism, should not be interpreted in isolation as evidence of active urinary tract infection or as an indication for antibiotic therapy. The clinical context remains essential for distinguishing symptomatic UTI from asymptomatic bacteriuria, particularly in frail older adults.

Our results confirm that clinical presentation was often non-specific. Although 83.3% of residents with positive urine cultures presented with urinary symptoms associated with systemic or non-specific signs, 16.7% had only non-specific geriatric manifestations (Table 3). Previous reports frequently misattribute confusion, falls, or functional decline to urinary tract infection, despite poor diagnostic specificity (8–10). In our study, these non-specific manifestations were not considered sufficient, in isolation, to establish symptomatic UTI. Rather, clinical findings were interpreted in conjunction with microbiological results, recognizing that bacteriuria and leukocyturia are frequent among nursing home residents and may reflect asymptomatic bacteriuria rather than active infection. Such presentations contribute to diagnostic uncertainty and promote unnecessary urine testing and antimicrobial initiation (11, 21).

**TABLE 3:** Clinical characteristics and empirical antibiotic therapy according to urine culture results.

Clinical characteristics	Negative urine culture (*n* = 27)	Positive urine culture (*n* = 60)	Total *n*	*p*-value
Acute urinary retention, n (%)				
No	22 (81.5%)	56 (93.3%)	78	0.18
Yes	5 (18.5%)	4 (6.7%)	9	
Urinary symptoms, n (%)				
Yes	11 (40.8%)	47 (78.3%)	58	0.001
No	16 (59.2%)	13 (21.7%)	29	
Atypical signs, n (%)				
Yes	27 (100.0%)	57 (95.0%)	84	0.55
No	0 (0.0%)	3 (5.0%)	3	
General signs, n (%)				
Yes	5 (18.5%)	47 (78.3%)	52	<0.001
No	22 (81.5%)	13 (21.7%)	35	
Empirical antibiotic therapy, n (%)				
No	11 (40.8%)	7 (11.7%)	18	<0.001
Yes	16 (59.2%)	53 (88.3%)	69	
Composite clinical presentation, n (%)				
Only non-specific geriatric manifestations	16 (59.2%)	10 (16.7%)	26	<0.001
Urinary symptoms with systemic or non-specific signs	11 (40.8%)	50 (83.3%)	61	

Microbiologically, Enterobacterales were the predominant pathogens, with Escherichia coli accounting for more than one-third of isolates (Figure 2). This distribution is consistent with epidemiological data from long-term care facilities (4, 5). However, the proportion of extended-spectrum β-lactamase-producing Enterobacterales (ESBL-E) was substantial, affecting 51.7% of positive urine cultures (Figure 3). In a French point-prevalence survey conducted across 18 nursing homes in the Franche-Comté region, Broussier et al. reported ESBL-E carriage in 19.8% of 262 screened residents, with prevalence varying from 0% to 43.8% across facilities (22). Direct comparison with our findings should be made cautiously because the denominators and study designs differ: our study reports the proportion of ESBL-E among positive urine cultures obtained from residents evaluated for suspected UTI, whereas Broussier et al. assessed ESBL-E carriage among nursing home residents through systematic screening. Nevertheless, the high proportion of ESBL-E identified in our urine cultures highlights the substantial burden of antimicrobial resistance in this selected population. Prior antibiotic exposure and recent hospitalization, both recognized risk factors for ESBL-E carriage in French nursing home residents, may partly contribute to this burden (22). However, the detection of an ESBL-producing organism does not, in itself, indicate active infection or justify antibiotic treatment, as resistant organisms may represent colonization or asymptomatic bacteriuria.

Antibiotic prescribing patterns further underline the issue of overtreatment. All residents with positive urine cultures received antibiotic therapy, including cases where symptoms were non-specific or where cultures were negative following empirical treatment (Table 3). Twenty-seven empirical treatments were initiated despite subsequent negative urine cultures, and 42.5% of antibiotic prescriptions were classified as inappropriate according to SPILF criteria. These results are in line with previous studies demonstrating that non-specific symptoms and urine test results often drive inappropriate antibiotic use in long-term care settings (19, 24, 25).

Fosfomycin was the most frequently prescribed antimicrobial agent in our cohort. This prescribing pattern is consistent with current French recommendations and the European Medicines Agency (EMA), which recognize oral fosfomycin trometamol as an appropriate first-line treatment for uncomplicated lower urinary tract infections because of its efficacy, convenient single-dose administration, favorable safety profile, and limited ecological impact compared with broader-spectrum antibiotics. These characteristics make fosfomycin particularly suitable for frail older adults. Furthermore, recent evidence has confirmed its clinical efficacy and safety, while microbiological studies have demonstrated sustained activity against many ESBL-producing Escherichia coli isolates, supporting its role as a valuable carbapenem-sparing option for susceptible multidrug-resistant urinary pathogens (27). This may partly explain clinicians’ preference for fosfomycin in our cohort, where 51.7% of positive urine cultures yielded ESBL-producing Enterobacterales. Nevertheless, our findings indicate that the principal issue was not necessarily the antibiotic selected but rather the initiation of antimicrobial therapy in residents who did not meet clinical criteria for symptomatic urinary tract infection (27).

Importantly, seven residents received antibiotics without urine culture because no urine sample could be obtained, reflecting a common clinical scenario in highly dependent patients. While pragmatic, such situations further increase the risk of diagnostic uncertainty and inappropriate antibiotic exposure. In these circumstances, the decision to initiate treatment should nevertheless remain based on the overall clinical presentation and established diagnostic criteria rather than on the inability to obtain a microbiological sample alone.

Overall, these findings support the concept that urine testing itself may act as a driver of antibiotic overuse. Indiscriminate use of dipsticks and cultures in frail older adults frequently leads to the detection of asymptomatic bacteriuria, which is then misinterpreted as infection, resulting in unnecessary antibiotic prescriptions (20, 21). This cascade effect has been widely described and represents a key target for antimicrobial stewardship interventions in nursing homes (14, 21, 22). However, our observational design does not allow us to conclude that urine testing directly causes antimicrobial resistance. Rather, unnecessary testing and subsequent antibiotic exposure may form part of a broader cycle of diagnostic uncertainty and antimicrobial use in which multiple factors contribute to the emergence and persistence of resistant organisms.

From a stewardship perspective, our results reinforce the importance of strict adherence to clinical criteria before ordering urine cultures. The Loeb minimum criteria remain a practical tool to reduce inappropriate testing and should be systematically implemented in long-term care facilities (21). Educational interventions targeting healthcare professionals, combined with diagnostic stewardship strategies, are essential to reduce unnecessary urine testing and antibiotic exposure (22). These interventions should emphasize that a positive urine culture, including the identification of multidrug-resistant organisms, does not necessarily represent symptomatic infection and should not automatically trigger antibiotic treatment.

Finally, the high proportion of ESBL-producing organisms observed among positive urine cultures highlights the substantial burden of antimicrobial resistance in this selected nursing home population. However, this finding should be interpreted cautiously. The retrospective observational design of our study does not allow causal conclusions regarding a relationship between urine testing practices, antibiotic prescribing, and antimicrobial resistance. Several potentially relevant confounding factors, including previous antibiotic exposure, recurrent urinary tract infections, urinary catheterization, prior hospitalization, and other healthcare-related exposures, may independently influence the risk of colonization or infection with ESBL-producing organisms. These factors were not systematically available or collected in sufficient detail to allow adjustment for their potential effects. Therefore, the 51.7% proportion of ESBL-producing isolates should be considered a descriptive finding in this specific study population rather than evidence of a direct causal relationship with urine testing practices.

The findings of this study should also be interpreted in light of their limited external validity. The relatively small sample size and inclusion of only two nursing homes may limit the generalizability of our results to other long-term care facilities with different resident characteristics, organizational structures, healthcare resources, or antimicrobial prescribing practices. Nevertheless, the observed patterns highlight clinically relevant opportunities for improving diagnostic stewardship in nursing homes. Future multicenter prospective studies including a larger number of facilities and systematically collected information on previous antibiotic exposure, urinary catheterization, recurrent infections, hospitalization, and other healthcare-related factors are needed to better characterize determinants of inappropriate antibiotic prescribing and antimicrobial resistance in this population.

## Conclusion

Inappropriate urine testing and antibiotic prescribing were frequent among nursing home residents evaluated for suspected UTI. Positive urine cultures, including those yielding ESBL-producing organisms, should not be interpreted in isolation as evidence of symptomatic infection or an indication for antibiotic therapy. Strengthening diagnostic stewardship and adherence to established clinical criteria may help reduce unnecessary urine testing and antimicrobial exposure while promoting appropriate antibiotic use.

## Data Availability

The raw data supporting the conclusions of this article will be made available by the authors, without undue reservation.
